# Axillary artery to left anterior descending coronary artery bypass with an externally stented graft: a technical report

**DOI:** 10.1186/1749-8090-3-6

**Published:** 2008-02-12

**Authors:** Thanos Athanasiou, Emmanouil I Kapetanakis, Christopher Rao, Loris Salvador, Ara Darzi

**Affiliations:** 1 Imperial College of Science, Technology and Medicine, Department of Surgical, Oncology and Technology, St. Mary's Hospital, London, UK; 2 Cardiothoracic Surgery Department, Treviso Ca' Foncello Hospital, Treviso, Italy

## Abstract

With the proliferation of minimally invasive cardiac surgery a number of alternative inflow sites for coronary artery bypass grafting have been utilized, especially in higher risk patients. The use of axillary-coronary artery bypass is a safe and effective alternative especially in the case of patients requiring redo coronary revascularization. However, the length and convoluted course of the axillary-coronary vein graft makes is susceptible to twisting, trauma and neointimal hyperplasia. We therefore report a case of an axillary-coronary artery bypass in a high risk patient in which a Dacron conduit was used to externally support and protect the vein graft to the left anterior descending artery. Surgical technique and considerations are presented and discussed.

## Background

The renewed interest in minimally invasive coronary artery bypass graft surgery without the use of cardiopulmonary bypass (MIDCAB) has produced various techniques utilizing alternative inflow sources for myocardial revascularization. These techniques are particularly applicable to high risk patients with a severely calcified thoracic aorta or in patients who require repeat coronary artery revascularization but in which the left internal mammary artery to anterior descending artery graft is occluded while other grafts are still patent. In these patients the potential risk to underlying vital structures during repeat sternotomy or mini sternotomy is higher and a minimally invasive anterior thoracotomy approach is preferable.

The use of the axillary artery as a site for the proximal graft anastomosis has been previously described in several single case reports [1,2] and small case series [3-9]. However, utilizing the axillary artery as an inflow vessel is technically challenging as the extrathoracic section of the graft makes it more susceptible to kinking and occlusion. We report a case of axillary artery to left anterior descending (LAD) graft surgery in which the graft was externally stented with a Dacron tube conduit and present the surgical technique utilized.

## Case presentation

A 64 year old patient was referred to our cardiothoracic surgery service with persistent symptoms of angina (NYHA Class: III). His previous medical history included type II diabetes mellitus managed with oral antiglycemic medication, hypertension and hypercholesterolemia. He had undergone a previous coronary artery bypass graft (CABG) surgery seven years ago with a left internal thoracic artery (LITA) anastomosed to the proximal LAD coronary artery, and two saphenous vein (SV) grafts to the proximal posterior descending artery (PDA) and an obtuse marginal (OM) branch. Despite a number of uneventful years his symptoms gradually returned. Repeat angiographic studies demonstrated a complete occlusion of the LITA, the SV to the OM graft was patent while the SV graft to the PDA graft had a 50% stenosis. Myocardial perfusion imaging demonstrated reduced perfusion and ischaemic myocardium primarily in the LAD perfusion area. Therefore given the risks of re-sternotomy a limited revascularization of the LAD territory through a minimally invasive surgical approach was decided upon.

### Surgical approach

**Figure 1:**
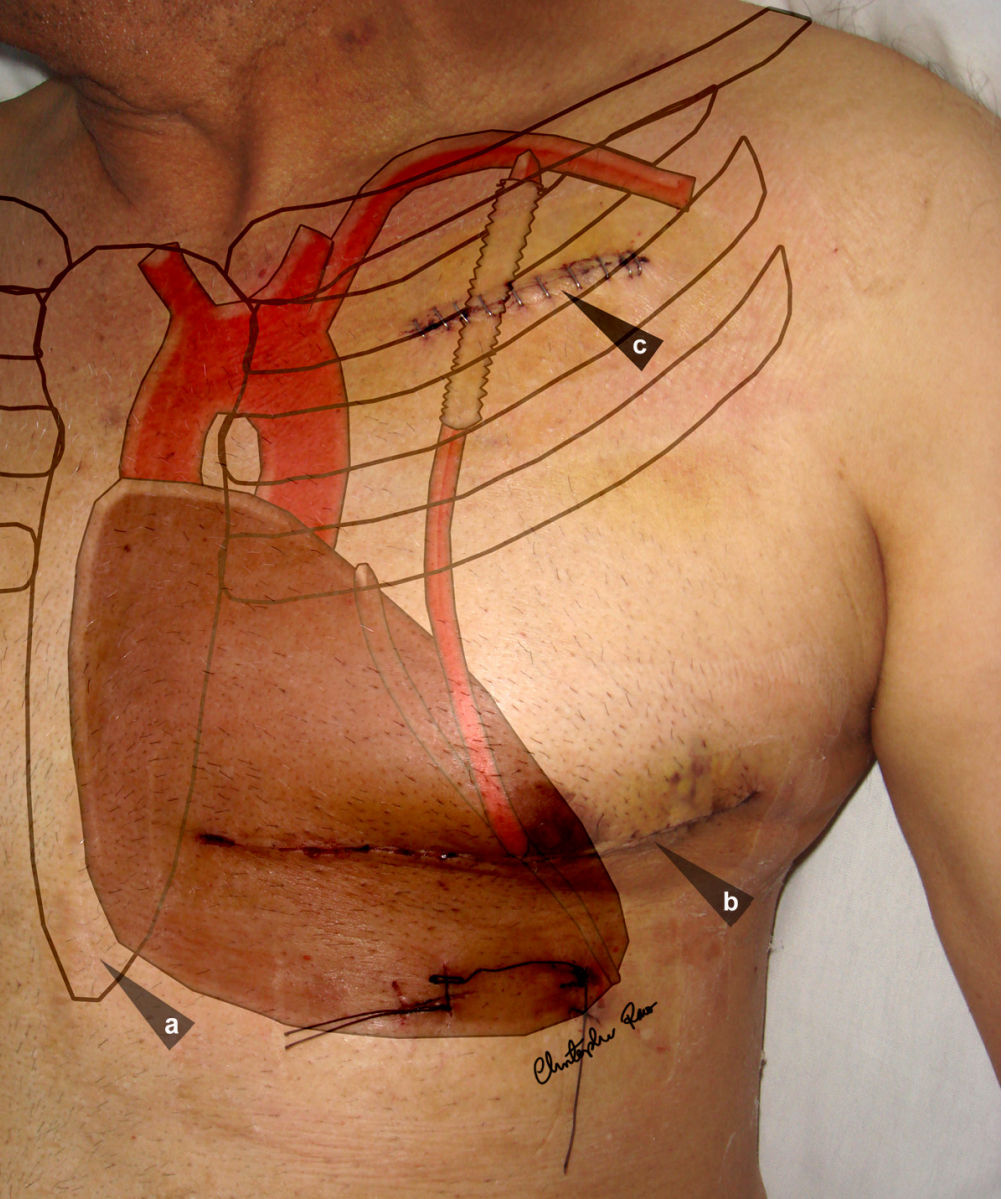
External anatomy and internal course of the externally stented axillary-coronary vein graft. (a: previous median sternotomy, b: anterior thoracotomy, c: subclavicular insicion).

A limited 6 cm anterior thoracotomy was performed at the level of the fifth intercostal space (Figure 1). Adhesions between the lung and the mediastinum and between the lung and the heart were then divided. The pericardium was incised longitudinally to expose and examine the distal LAD for its suitability for grafting. Its size and degree of calcification were assessed as adequate. A second 2.5 cm short transverse incision was made below the left clavicle to expose the axillary artery (Figure 1). Concurrently a 20 cm SV graft was harvested from the right leg. After systemic heparinization the SV graft and the left axillary artery were anastomosed end to side with a 6.0 propylene suture. A 10 mm Thoracoport tunneling device (Autosuture, Norwalk, CT) was used to create a channel over the rib and to make an opening into the superior-medial aspect of the first intercostal space. A 6 mm Dacron tube conduit (Boston Scientific Corp, Natick, MA) was positioned over the vein graft to reinforce it and prevent kinking. The relative length of conduit to vein was approximately 50% with the conduit covering primarily the graft through its intrathoracic course. The proximal end of the Dacron conduit was then sutured on the intercostal muscle to secure it. The combined graft and conduit was then passed inferiorly to the axillary vein and through the opening into the thoracic cavity (Figure 1). The graft was then retrieved from the apex of the pleural cavity using a long curved clamp, taking care not to twist or kink it. Next the target LAD site was dissected and occluded proximally and distally with two 3.0 Gore-Tex (W.L. Gore and Associates, Flagstaff, AZ) vascular snares. Finally an end to side SV-LAD anastomosis was completed with a running 7.0 propylene suture. Stabilization of the target vessel was achieved with the Octopus (R) 4 tissue stabilizer (Medtronic Inc, Minneapolis, MN). Total occlusion time of the LAD was 7 minutes and total revascularization time (from skin incision to end of anastomosis) was 90 minutes.

### Patient follow up

The patient's postoperative course was uneventful and was discharged home after five days. At six week and three month follow up the patient was well and had returned to normal activity. The patient refused follow up angiography at three months, but it is planed to be performed after a year. Dobutamine stress echo demonstrated good function at the LAD territory.

## Comments

A number of authors have previously described their experience with axillary-coronary artery bypass grafting using a saphenous vein conduit. Coulson and Knight were the first to publish within months of each other, about cases in which an SV graft was connected to the LAD artery using the left axillary or subclavian artery as an inflow source [1,2]. Both surgeons appear to have developed this technique independent of each other and whereas Knight placed the graft behind the pectoral muscles, superficial to the ribs, Coulson utilized an intrathoracic approach. Similarly, Tovar et al, used a SV graft anastomosed to the axillary artery passing it into the thorax through an opening made by resecting the proximal part of the first rib [10].

Since then a total of 125 cases have been reported either individually or as case series in the literature [9]. Bonatti and the Innsbruck group have undertaken both anatomical and animal studies on the feasibility and efficacy of axillary-coronary artery bypass, culminating in the presentation of their case series [5,11,12].

The primary cause for application of the technique in patients, were problems with the internal thoracic artery during a MIDCAB procedure, as in Coulson's case in which previous sternal wiring had rendered the LITA unusable or less often due to post-irradiation changes as in the case reported by Wolf et al, [1,6]. The utilization of the technique for the management of a severely atherosclerotic ascending aorta has also been suggested [13]. However, this use still remains controversial as the innominate and internal thoracic arteries represent alternatives that do not require additional incisions or an intrapleural course for the conduit. But as the innominate artery is often involved in the atherosclerotic process precluding the application of a tangential clamp, the use of the axillary artery as an inflow vessel is becoming more widespread. A major advantage of using the axillary artery is that it is rarely the site of significant atherosclerosis and if any embolization does occur the brain and kidneys are spared [5].

Finally, the use of axillary-coronary artery bypass has been advocated in high risk patients who had a previous cardiac operation [5]. Re-sternotomy carries a significant risk of operative mortality and morbidity, especially in unstable, multimorbid patients with impaired ventricular function [14]. In such cases axillary-coronary artery bypass via a mini thoracotomy may be the only surgical option available.

As with all novel procedures there have been a number of variants in technique; enough to prompt the suggestion of a classification by conduit type, with the principal variation being the course of the graft and the way it is routed into the thoracic cavity [15]. There has been much discussion about whether the graft should be placed within the thoracic cavity or outside. Tovar and others have advocated an intrathoracic route and have expressed concern about Knight's tunneling of the graft supracostaly behind the pectoralis major muscle [10,15]. However, Shabb and Khalil have reported excellent patency in a three patient case series using the supracostal technique [4].

In our case we utilized a by Coulson's classification "type F conduit", routing the graft inside the chest through an incision at the first intercostal space. We believe that this course has two advantages; first pulmonary expansion exerts less pressure on the graft than muscle contraction and secondly the length of vein required is shorter, which can be an issue in patients, like this one, in which significant saphenous vein segments were used up in a previous CABG surgery. However, the alterantive supracostal route advocated by Knight and colleagues does have one advantage, which is that it allows for non-invasive Doppler ultrasound assessment and follow up of graft patency [9].

Another controversial aspect of the technique involves the point of entry of the graft into the thoracic cavity. Some authors have recommended partial resection of the first or second rib so as to allow for an unimpeded course and to avoid mechanical trauma and the development of neointimal proliferation [1,10]. However, resection of the first or second rib might cause chest wall instability especially when combined with fourth rib resection to expose the LAD [10]. In our case we utilized a wide incision, large enough to allow the insertion of two fingers as Magovern has previously recommended, in the medial aspect of the first intercostals space to prevent compression of the graft as it enters the thoracic cavity [8].

Because of the small number of cases that have been reported thus far there has been little information about patency rates of axillary-coronary artery bypass. Coulson et al, reported a one-year patency rate of 80% in 10 patients operated by this technique [3]. Similarly, Bonatti et al, reported a vein graft patency rate of 13/14 (93%) at a mean follow up of seven months [5].

As the length of the vein graft required is significantly longer than for conventional aortic-coronary artery bypass and due to its crossing of several muscular and bony anatomical structures such as the first rib, the intercostal space, the pleural cavity and the pericardium the amount of mechanical trauma and subsequent development of neointimal hyperplasia would theoretically be increased. Early histologic changes with endothelial defects, intraluminal deposition of fibrin, mural edema, smooth muscle cell necrosis in the media and inflammatory infiltrates have been observed in animal studies of axillary-coronary artery bypass [12]. Similarly, Angelini et al have noted intimal thickening and medial expansion during the first week after SV grafting into the carotid artery in a porcine model [16].

Therefore, Bonatti and other authors have expressed concern about the course of the graft and especially about the development of microtrauma and early degenerative changes at the rib crossing site, even though so far studies have indicated there is no predilection for neointimal hyperplasia to develop in the area of the thoracic window but rather seems to develop diffusely along the whole length of the graft [15].

In an attempt to reduce graft trauma we utilized a Dacron conduit as an external stent to protect and reinforce the graft and minimize the potential risk of kinking or distortion as it enters the chest cavity through the intercostal space and as it turns and passes from the anterior to the posterior surface of the lung. It was hypothesized that the Dacron conduit would act as a mechanical shock absorber and thus reduce traumatic contact by the vein. Therefore, the graft was reinforced from its proximal end to approximately its mid section for the most part of its intrathoracic route. To facilitate entry into the pericardial cavity and reduce increased compression of the vein graft, the graft was "naked" in its distal portion.

By using a conduit with a slightly larger diameter than the graft we allowed for expansion of the vein without strangulating it while at the same time minimizing stretching and distention. Animal studies on external stenting have demonstrated that graft failure is the result of medial and intimal thickening caused by medial vascular smooth muscle migration and proliferation of extracellular matrix deposition [17]. Vein graft wall thickening may represent an adaptation to the hemodynamic changes in pressure and flow [17,18]. The increased intraluminal pressure of the arterial circulation causes the walls of the vein to stretch and distend which then produces neointimal hyperplasia, occlusion and graft failure [18]. Therefore, by utilizing external stenting we hope to reduce the mechanical stress on the axillary-coronary vein graft in addition to reinforcing its tortuous path. As far as we are aware this is the first time in which an externally stented vein graft has been used for axillary-coronary bypass surgery. So far the follow up in our patient has been encouraging but obviously further study is required to establish the efficacy of this technique.
